# Gut microbiota-derived tryptophan metabolites alleviate liver injury via AhR/Nrf2 activation in pyrrolizidine alkaloids-induced sinusoidal obstruction syndrome

**DOI:** 10.1186/s13578-023-01078-4

**Published:** 2023-07-08

**Authors:** Haitao Shang, Chao Huang, Zhuanglong Xiao, Pengcheng Yang, Shengyan Zhang, Xiaohua Hou, Lei Zhang

**Affiliations:** 1 Division of Gastroenterology, Union Hospital, Tongji Medical College, Huazhong University of Science and Technology, Wuhan, 430022 China; 2 Department of Gastroenterology, Qingdao Hospital, University of Health and Rehabilitation Sciences (Qingdao Municipal Hospital), Qingdao, 266071 China

**Keywords:** Hepatic sinusoidal obstruction syndrome, Pyrrolizidine alkaloids, Gut microbiota, Tryptophan metabolism, Aryl hydrocarbon receptor, Nuclear factor erythroid 2-related factor 2

## Abstract

**Background and aims:**

Hepatic sinusoidal obstruction syndrome (HSOS) is caused by toxic injury, such as pyrrolizidine alkaloids, to the liver sinusoidal endothelial cells, and the gut microbiota may be involved. However, the specific role and underlying mechanism of gut microbiota in HSOS is unknown.

**Methods:**

HSOS model was established by gavage of monocrotaline (MCT) in rats. Fecal microbiota transplantation (FMT) with HSOS-derived or healthy gut flora was also conducted to validate the role of gut microflora in MCT-induced liver injury. The microbial 16 s rRNA analysis and untargeted metabolomics analysis in the faeces were performed to identify HSOS-related flora and metabolites. Finally, by supplementation with specific tryptophan metabolites, such as indole-3-acetaldehyde (IAAld) and indole acetic acid (IAA), we further confirmed the role of tryptophan metabolism in HSOS and the role of the AhR/Nrf2 pathway in MCT-induced liver injury.

**Results:**

MCT induced HSOS-like liver injury in rats with significantly altered gut microbiota. Particularly, some tryptophan-metabolizing bacteria reduced in MCT-treated rats, such as *Bacteroides*, *Bifidobacterium*, *Lactobacillus* and *Clostridium*, and accompanied by a decrease in microbial tryptophan metabolic activity and a series of tryptophan derivatives. Restoring the gut microbiota via FMT improved MCT-induced liver damage, while HSOS-derived gut microbiota aggravated the liver injury induced by MCT. Supplementation with microbial tryptophan derivatives (IAAld or IAA), or 6-formylindolo(3,2-b)carbazole (Ficz, an AhR agonist) could activate the AhR/Nrf2 signaling pathway, thereby attenuating the MCT-induced liver oxidative stress and liver sinusoidal endothelial cells injury.

**Conclusions:**

Gut microbiota plays a critical role in MCT-induced HSOS, with inadequate microbial tryptophan metabolism in the gut and consequently a lower activity of the AhR/Nrf2 signaling pathway in the liver, which should be a potential target for the management of HSOS.

**Supplementary Information:**

The online version contains supplementary material available at 10.1186/s13578-023-01078-4.

## Introduction

Hepatic sinusoidal obstruction syndrome (HSOS), also known as hepatic veno-occlusive disease (HVOD), is rare but life-threatening vascular liver disease with typical performance of painful hepatomegaly, ascites and jaundice [1, 2].

HSOS is characterized by toxic injury to small hepatic vessels, particularly the sinusoidal endothelium [3]. The cytoreductive therapy prior to hematopoietic stem cell transplantation and oxaliplatin-containing adjuvant chemotherapy are the main cause of HSOS in the developed countries [4]. However, in China, the primary cause of HSOS may be intake of pyrrole alkaloids (PAs)-containing herbal medicines or dietary supplements [5, 6], especially for *gynura segetum* (Tusanqi) which is a traditional Chinese herbal medicine rich in PAs [2, 3]. The toxic destruction of sinusoidal endothelial cells by PAs metabolites should be an accepted initial event, while our knowledge for HSOS is far from enough.

Gut microbiota has been considered an important player for the development of various acute and chronic liver diseases, including non-alcoholic/alcoholic liver disease, hepatitis virus infection, primary sclerosing cholangitis and liver cirrhosis, as well as the chemical/drug-induced liver injury [7–9]. Hepatotoxicity is likely induced by gut dysbiosis, mucosal barrier damage, systemic immune activation, microbial-associated molecular patterns and microbial metabolites [10, 11]. In the previous study, it indicated that gut dysbiosis cooperates with retrorsine, a representative PAs, to promote HSOS in mice via disrupting the gut barrier, both the intestinal epithelial barrier and gut-vascular barrier (GVB) [12]. The leaky gut could drive the hepatic injury by overloaded exposure to enteric antigens and toxins, even bacterial translocation [13]. Meanwhile, lack of some gut-derived microbial metabolites which have key beneficial effects could also lead to liver dysfunction, such as tryptophan derivatives, short-chain fatty acids, secondary bile acids, etc [14]. For instance, the microbial tryptophan metabolism can induce the activation of aryl hydrocarbon receptor (AhR) and improve the alcohol-induced liver injury [15]. However, in PAs-induced HSOS, the specific role of gut flora and microbial metabolism needs to be further elucidated.

Consequently, in the present study, we aimed to identify the HSOS-related microbiota and metabolites in the gut of monocrotaline (MCT)-induced HSOS model via 16 s rRNA sequencing and untargeted metabolomics. Accordingly, the specific microbial metabolites and potential molecular targets will be further explored and validated in the process of HSOS.

## Materials and methods

### Animals

Male Sprague Dawley (SD) rats (aged 5–6 weeks, weighed 160–180 g; VitalRiver Bioscience, Beijing, China) were used. All experimental rats were housed in specific pathogen-free (SPF) condition at a controlled room temperature (22 ± 1 °C) with a light/dark cycle of 12:12 h, and free access to a standard laboratory diet and water. All animal experiments were approved by the Animal Care and Use Ethics Committee, Tongji Medical College, HUST, Wuhan, China.

### Monocrotaline-induced HSOS model and treatment

Monocrotaline (MCT, a representative PAs) -treated rat is generally accepted as a classic experimental model of HSOS [16, 17]. Briefly, after fasting for 12 h, the rats were orally administered with MCT (90 mg/kg; Sigma Aldrich) for once. The histopathological changes of the liver are similar with those in human HSOS at 48 h after MCT (90 mg/kg) treatment in rats, with hepatic sinusoid dilatation and congestion, hepatocyte coagulation necrosis, and destroyed central lobular vein endothelium [18]. Additionally, the liver injury induced by different doses of MCT at 45 mg/kg, 60 mg/kg, 75 mg/kg, 90 mg/kg was further evaluated, which indicated that the HSOS-like liver injury began to be significant at the dose of 60 mg/kg.

To confirm the role of gut microbiota in the process of PAs-induced HSOS, the fecal microbiota transplantation (FMT) experiments were performed. The fecal bacteria suspension with the donor feces form HSOS rats (FMT-HSOS) or Normal rats (FMT-Norm) was prepared. The donor feces were dissolved in PBS (2 g/5 ml) and filtered twice with double-layer gauze to obtain the fresh fecal bacteria suspension. The experimental scheme: ①To evaluate the therapeutic action of normal gut microbiota on MCT-induced HSOS, the MCT (60 mg/kg)-treated rats were treated with FMT-Norm once daily for 5 consecutive days following MCT administration, while PBS was conducted as vehicle control. ② To clarify whether the HSOS-related gut microbiota could lead to similar liver injury, the FMT-HSOS was performed on healthy rats by gavage once a day for 7 days, while the FMT-Norm was conducted as control. Before FMT, rats were given a cocktail of antibiotics (100 mg/kg vancomycin, 200 mg/kg neomycin, 200 mg/kg metronidazole and 200 mg/kg ampicillin) by gavage once daily for 7 days to deplete the gut microbiota [19, 20]. ③To further verify the role of HSOS-related gut microbiota in MCT-induced liver injury, the rats were given cocktail of antibiotics for 7 days to deplete the gut microbiota, then pretreated FMT-HSOS or FMT-Norm by gavage one a day for 7 days, and following with one MCT (60 mg/kg or 90 mg/kg) administration, and final sacrificed after 48 h for test.

To investigate the role of microbial tryptophan metabolism in PAs-induced HSOS, the tryptophan derivatives such as indole acetaldehyde (IAAld) and indole-3-acetic acid (IAA) were selected based on metabolomic analysis, which are natural ligands for AhR. The rats were pretreated with exogenous IAAld (20 mg/kg; TargetMol, Chnia) or IAA (20 mg/kg; TargetMol, Chnia) by gavage once a day for 5 days before MCT administration, while AhR agonist 6-formylindolo(3,2-b)carbazole (Ficz, 1.5 μg/rat; MedChemExpress, China) was used as a positive control, and PBS was conducted as vehicle control. All rats were sacrificed 48 h after MCT treatment, and the blood, feces, and liver samples were collected.

### Primary liver sinusoidal endothelial cell culture and treatment

A standard rat primary liver sinusoidal endothelial cells (LSECs) were used for in vitro study (RA-6017, Cell Biologics, Chicago, USA). The LSECs were cultured in complete rat endothelial cell medium (M1266, Cell Biologics, Chicago, USA). The LSECs at passage 4 to 6 were used for following experiments. Before that, the fecal supernatants from HSOS rats (HSOS-FSN) and normal rats (Norm-FSN) were prepared. In brief, feces were dissolved and homogenized in PBS solution (1 g/5 ml), and then the supernatants were collected after centrifugation (5000 rpm, 4 °C, 10 min) filtered through a 0.22 μm-sized filter, and stored at − 80 °C. The LSECs were seeded in standard 12-well plates and incubated with 200 μl HSOS-FSN or 200 μl Norm-FSN in 2 ml culture medium for 48 h, before which the LSECs were pretreated with 200 μM Ficz (MedChemExpress, China) for 24 h, while PBS was conducted as vehicle control. At the end of intervention, cell viability was measured via a commercial cell counting kit-8 (CA1210, Solarbio, Beijing, China), and was normalized as the percentage of control. Finally, the LSECs were collected and used for mRNA and protein quantification via Real-time PCR and western blot analysis.

### 16S rRNA sequencing and metabolomics analysis

Fecal microbial DNA was extracted from stool samples and then used for 16S rRNA sequencing on an Illumina MiSeq platform (Illumina, USA). Quantitative Insights into Microbial Ecology (QIIME) was used to demultiplex sequence and filter low-quality reads following the criteria. The microbial α diversity was determined by the chao1 index, and β diversity was assessed by unweighted UniFrac distance matrics and visualized by principal coordinate analysis (PCoA). The microbial composition was then analyzed on phylum and genus level, and the relative abundance of specific genera that are known tryptophan-metabolizing bacteria were further identified, such as *Bacteroides*, *Bifidobacterium*, *Lactobacillus*, *Clostridium* and *Prevotella_9*. Finally, the Phylogenetic Investigation of Communities by Reconstruction of Unobserved States (PICRUSt) was performed to predict the metagenome functional profiles from 16S rRNA surveys according to the Kyoto Encyclopedia of Genes and Genome (KEGG) orthology database [21].

Untargeted metabolic profiling based on liquid chromatography-mass spectrometry (LC–MS) was performed (TreatGut Bio, Xiamen, China) [22]. Statistical analysis was conducted on log2-transformed metabolite values. Orthogonal partial least squares-discriminant analysis (OPLS-DA) was used for variables with less correlation. The fold change and VIP value of the OPLS-DA model were combined to screen different metabolites. The KEGG orthology database was used for annotation of the different metabolites as described previously [23].

### Liver histological analysis

Fresh liver tissues were fixed in 4% paraformaldehyde for 24 h, then embedded in paraffin, and subsequently sectioned at 4 μm. The slides were stained with hematoxylin and eosin (H&E), and then analyzed blindly by a liver pathologist. The histological evaluation of HSOS were based on sinusoidal dilatation, coagulative necrosis of hepatocytes, central vein endothelial damage, and sinusoidal hemorrhage [16, 17]. Each of these features was graded on a 4-point scale: 0 = absent; 1 = mild; 2 = moderate; 3 = severe. Then, each score is added to get the total HSOS score.

As for the necrosis area,10 images are randomly selected in each low-power field of view (magnification 100 ×) and measured by Image J (National Institutes of Health).

### Western blot analysis

Total proteins were harvested from cells or liver tissues with RIPA Lysis Buffer (Beyotime, Shanghai, China) supplemented with phenylmethyl sulfonyl fluoride (PMSF) protease inhibitor and phosphatase inhibitor. A nuclear protein extraction kit (Solarbio, Beijing, China) was used to separate cytoplasmic and nuclear proteins. The corresponding protein concentration was determined by Pierce™ BCA Protein Assay Kit (Thermo Fisher, Massachusetts, USA). The denatured protein samples with appropriate protein amount were transferred to PVDF membrane after sodium dodecyl sulfate polyacrylamide gel electrophoresis (SDS-PAGE). The membranes were then blocked with 5% skimmed milk for 1 h and incubated overnight with specific antibodies against MMP-9 (ab76003, Abcam), AhR (GTX22770, GeneTex), CYP1A1(GTX55582, GeneTex), Nrf2 (PA5-88084, Thermofisher), Lamin B1(YT5180, Immunoway), and GAPDH (ANT325, Antgene) in a shaker at 4 ℃. After washing and secondary antibody incubation (ANT019/020, Antgene), the protein bands were visualized by the FluorChem Imaging System (ProteinSimple, California, USA) using the commercial Pierce™ Fast Western Blot Kit (Thermo Fisher, Massachusetts, USA).

### Real-time PCR analysis

Total RNA was extracted from livers by using TRIzol reagent (Invitrogen, California, USA). The cDNA was synthesized and Quantitative Real-time PCR was performed in a Roche LightCycler R480 (Roche, Switzerland). The result was evaluated by the 2^−ΔΔCt^ method and expressed as the ratio of the control. The primer sequences involved in this study are provided in Additional file 2: Table S1.

### Serum biochemistry analysis

Fresh blood was collected and stored at room temperature for 1 h. Serum was then obtained by centrifugation at 850 g for 15 min. Serum alanine aminotransferase (ALT) and aspartate aminotransferase (AST) activity were determined with an automatic biochemical analyzer (HITACHI 7080, Japan).

### Measurement of liver oxidative stress levels

The levels of liver lipid peroxidation (LPO), reactive oxygen species (ROS), glutathione/oxidized glutathione (GSH/GSSG) and glutathione S-transferase (GST) in the liver tissues were detected. Specifically, liver tissues were collected in cold phosphate buffered saline (PBS). (1) As an indicator of LPO, malondialdehyde (MDA) was measured using a commercial kit (A003-1, Jiancheng Bio, Nanjing, China) and expressed as nmol per mg protein. (2) Hepatic ROS level was measured as described in a previous paper [24] and a commercial reactive oxygen species assay kit was used (CA1410, Solarbio, Beijing, China). The result was calculated in fluorescence units per mg protein and expressed as a percentage of control (% of control). (3) The contents of liver GSH and GSSG were determined using a Total GSH/GSSG assay kit (A061-1, Jiancheng Bio, Nanjing, China), then the ratio of GSH/GSSG was calculated. (4) The activity of liver GST was determined according to manufacturer's recommended procedures (SN101, Solarbio, Beijing, China) and expressed as U per mg protein.

### Statistical analysis

All data were displayed as mean ± SEM. For comparison of two groups, the two-tailed Student’s t test was used. Multiple comparisons were evaluated statistically by one-way analysis of variance (ANOVA) and post hoc Tukey test. P < 0.05 was considered as statistically significance. Data were statistically analyzed using the SPSS package, version 20.0 (SPSS Inc., Chicago, USA). The statistical graphs were created via the GraphPad Prism package, version 8 (GraphPad, California, USA).

## Results

### MCT induce HSOS-like liver injury in rats

**Fig. 1:**
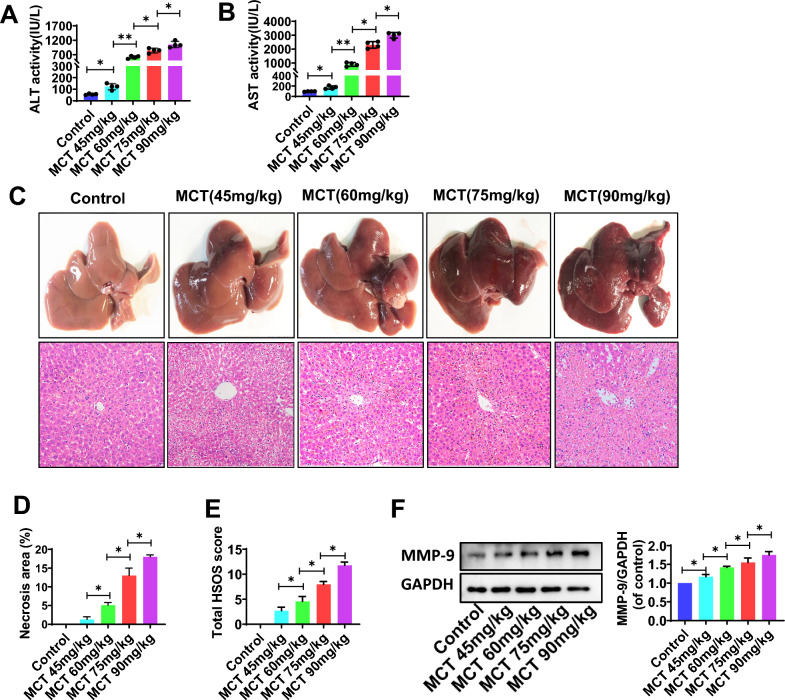
MCT induce HSOS-like liver injury in rats. **A, B** Serum ALT and AST activity. **C** The typical macroscopic (upper) and histological (down) observations of MCT-induced liver injury. **D** The % area of necrosis quantified in a low power field (100 ×). **E** The total HSOS score. **F** The protein expression of MMP-9 in liver tissues. GAPDH was used as a loading control. Data were shown as mean ± SEM. N = 4. *p < 0.05, **p < 0.01. MCT, monocrotaline; HSOS, hepatic sinus obstruction syndrome; ALT, alanine aminotransferase; AST, aspartate aminotransferase; MMP-9, matrix metalloproteinase-9; GPADH, glyceraldehyde-3phosphate dehydrogenase

MCT (90 mg/kg)-treated rats showed apparent liver injury and used as a classic experimental model of HSOS. It appeared typical liver histological changes of human HSOS, with hepatic sinusoid dilatation and congestion, hepatocyte coagulation necrosis, and destroyed central lobular vein endothelium, as we shown in Fig. 1C–E. Moreover, HSOS rats were also confirmed by elevated serum ALT (Fig. 1A) and AST (Fig. 1B) activities, and increased levels of hepatic matrix metalloprotein-9 (MMP-9, Fig. 1F), an injury marker of liver sinusoidal endothelial cells. Then, the liver injury induced by different doses of MCT from 45 mg/kg to 90 mg/kg was further evaluated. It indicated that MCT-induced hepatic sinusoid damage and hepatocyte necrosis is dose-dependent, and the histological changes began to be significant at the dose of 60 mg/kg, as well as the levels of hepatic MMP-9 and serum ALT and AST.

### HSOS-derived gut microbiota aggravates the liver damage induced by MCT

**Fig. 2:**
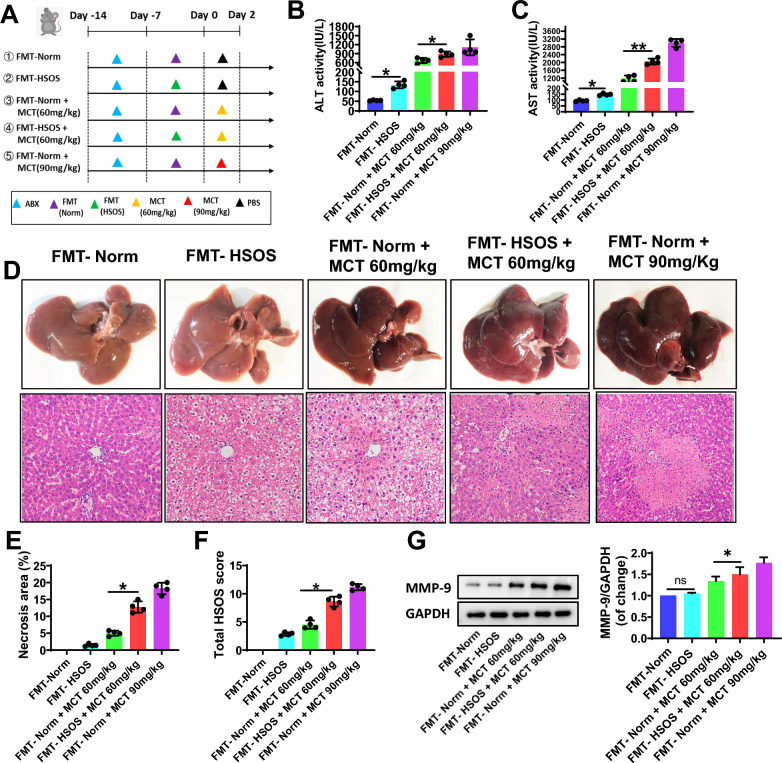
HSOS-derived gut microbiota aggravated the liver damage induced by MCT. **A** The study protocols. FMT-HSOS indicates fecal microbiota transplantation from HSOS rats, and FMT-Norm indicates fecal microbiota transplantation from normal rats. **B, C** Serum ALT and AST activity. **D** The typical macroscopic (upper) and histological (down) observation of liver injury. (E) The % area of necrosis quantified in a low power field (100 ×). **F** The total HSOS score. **G** The protein expression of MMP-9 in liver tissues. GAPDH was used as a loading control. Data were shown as mean ± SEM. N = 4. *p < 0.05, **p < 0.01. MCT, monocrotaline; HSOS, hepatic sinus obstruction syndrome; FMT, fecal microbiota transplantation; PBS, phosphate buffered saline; ALT, alanine aminotransferase; AST, aspartate aminotransferase; MMP-9, matrix metalloproteinase-9; GPADH, glyceraldehyde-3phosphate dehydrogenase

HSOS-derived gut microbiota may directly lead to the damage to the liver, although this effect was relatively mild. As it shown, compared with the control rats, the rats receiving FMT from HSOS rats (the FMT-HSOS group) developed mild liver injury (Fig. 2), based on the histological evaluation with higher % area of necrosis and total HSOS scores (all P < 0.05, Fig. 2D–F), accompanied by higher serum levels of ALT and AST (all P < 0.05, Fig. 2B, C). In addition, compared with the control rats, the abundance of Bacteroides (p = 0.045), Bifidobacterium (p = 0.047), Lactobacillus (p = 0.043) and Clostridium (p = 0.045), which known tryptophan-metabolizing bacteria, decreased in the FMT-HSOS group (Additional file 1: Fig. S1). Furthermore, HSOS-derived gut microbiota increased the susceptibility to MCT-induced liver damage. Relative to the rats accepted FMT from normal rats (FMT-Norm), the rats receiving FMT from HSOS rats (FMT-HSOS) showed more serious liver injury on exposure to MCT (60 mg/kg). The latter demonstrated more obvious hepatic sinusoid damage and hepatocyte necrosis in (Fig. 2D), with increased % area of necrosis (P = 0.044, Fig. 2E) and higher total HSOS score (P = 0.047, Fig. 2F), as well as significantly increased activity of serum ALT and AST (P < 0.05, Fig. 2B, C), and increased expression of hepatic MMP-9 (P < 0.05, Fig. 2G).

### Restoring the gut microbiota improves the liver injury induced by MCT

**Fig. 3:**
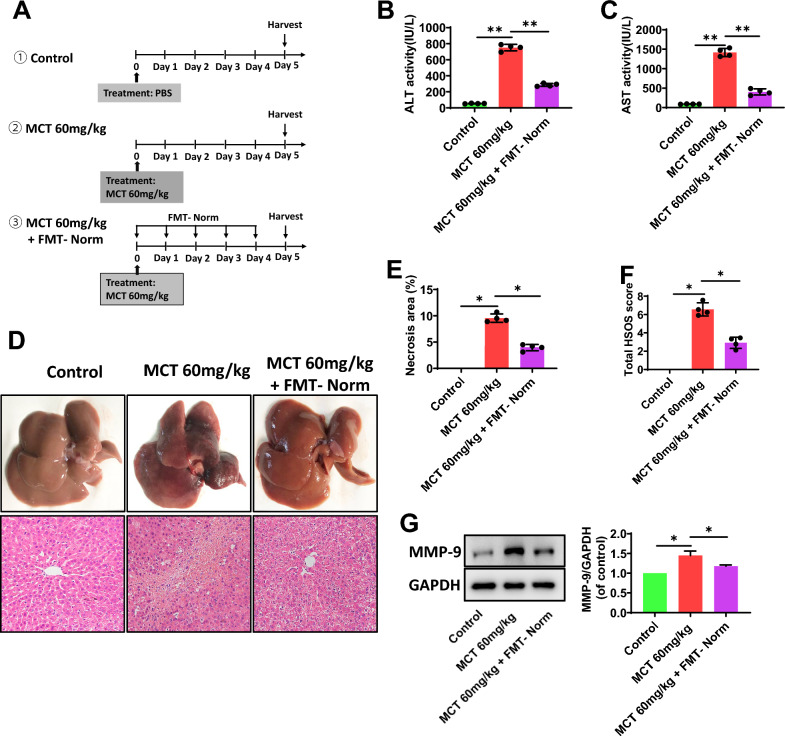
Restoring the gut microbiota improve the liver injury induced by MCT. **A** The study protocols. FMT-Norm indicated fecal microbiota transplantation from normal rats. **B, C** Serum ALT and AST activity. **D** The typical macroscopic (upper) and histological (down) observation of liver injury. **E** The % area of necrosis quantified in a low power field (100 ×). **F** The total HSOS score. **G** The protein expression of MMP-9 in liver tissues. GAPDH was used as a loading control. Data were shown as mean ± SEM. N = 4. *p < 0.05, **p < 0.01. MCT, monocrotaline; HSOS, hepatic sinus obstruction syndrome; FMT, fecal microbiota transplantation; PBS, phosphate buffered saline; ALT, alanine aminotransferase; AST, aspartate aminotransferase; MMP-9, matrix metalloproteinase-9; GPADH, glyceraldehyde-3phosphate dehydrogenase

FMT with healthy fecal microbiota (FMT-Norm) was conducted to normalize the intestinal flora in MCT (60 mg/kg)-treated rats (Fig. 3A and Additional file 1: Fig. S2). FMT-Norm treatment markedly reduced the activities of serum ALT (p = 0.007, Fig. 3B) and AST (p = 0.006, Fig. 3C) induced by MCT in rats. Also, FMT-Norm treatment reversed the MCT-induced hepatic histological injury, specifically alleviated the hepatic sinusoid dilatation and congestion, central lobular vein endothelial injury and hepatocyte coagulation necrosis (Fig. 3D), with decreased % area of necrosis and total HSOS scores (P < 0.01, Fig. 3E, F). The MCT-induced destroy of liver sinusoidal endothelial cells, such as hepatic MMP-9 expression, was effectively improved by FMT-Norm (Fig. 3G).

All above, it further confirmed that the gut microbiota may play a critical role in the process of pyrrolizidine alkaloids-induced HSOS.

### Features of gut microbiota in MCT-induced HSOS rats

**Fig. 4:**
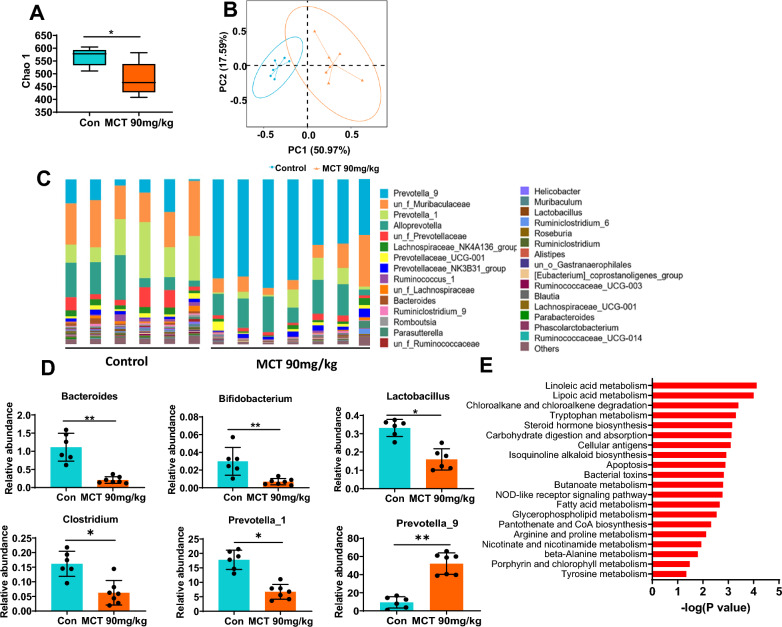
Features of gut microbiota in MCT-induced HSOS rats. **A** The alpha diversity (Chao1 index). **B** The PCoA plot analysis with unweighted UniFrac distance. **C** The bacterial composition at the genus level. **D** Changes of the specific genera in MCT-induced HSOS rats, especially the tryptophan-metabolizing bacteria. **E** The predicted bacterial functional genome based on KEGG database. Data were shown as mean ± SEM. N = 6–7. *p < 0.05, **p < 0.01. MCT, monocrotaline; HSOS, hepatic sinus obstruction syndrome; PCoA, principal coordinate analysis; KEGG, Kyoto Encyclopedia of Genes and Genomes

The fecal bacterial profiles in HSOS rats were significantly different from the healthy controls. The alpha diversity, such as the Chao1 index, was markedly decreased in HSOS rats than control rats (Fig. 4A). The PCoA plot further indicated that the microbial structure in HSOS rats was different absolutely from control rats (Fig. 4B). The bacterial composition was also markedly altered in the HSOS rats in comparison with the normal rats, especially at the genera levels (Fig. 4C). It showed decreased abundance of *Bacteroides* (p = 0.007), *Bifidobacterium* (p = 0.008), *Lactobacillus* (p = 0.043) and *Clostridium* (p = 0.045), while increased abundance of *Prevotella_9* (p = 0.009) in HSOS rats (Fig. 4D). Of note, these genera are known tryptophan-metabolizing bacteria and could produce a great deal of tryptophan derivatives which may act as the AhR ligands [25]. Moreover, we further identified several metabolic pathways that significantly altered in MCT-induced HSOS rats via the predicted bacterial functional genome based on KEGG database, in which the tryptophan metabolic pathway was markedly changed and less enriched in HSOS rats (Fig. 4E).

### Altered microbial tryptophan metabolism in MCT-induced HSOS rats

**Fig. 5:**
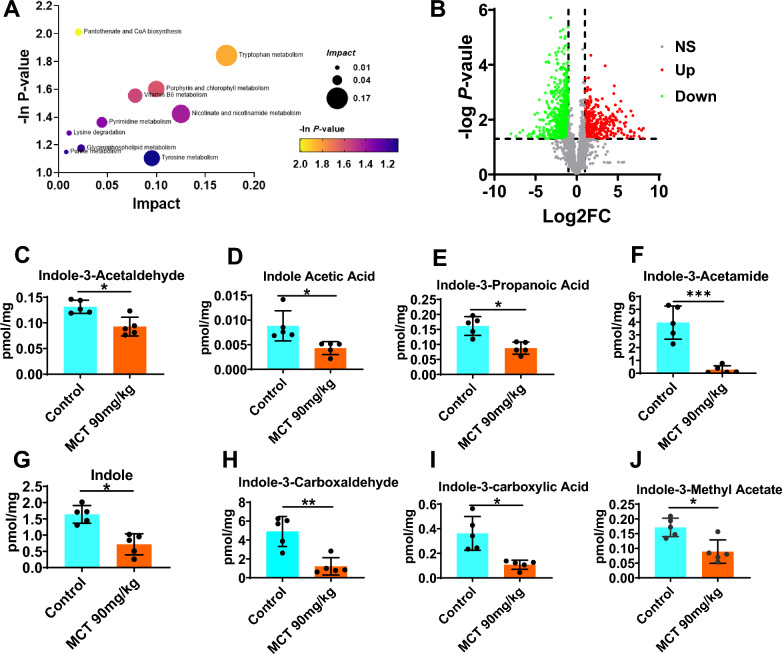
Altered microbial tryptophan metabolism in MCT-induced HSOS rats. **A** Bubble chart of metabolite pathway enrichment analysis based on KEGG pathway. The size of the bubble reflected the impact in the topology analysis, and the color of the bubble reflected the -ln P-value of the enrichment analysis. The lighter the color, the smaller the P value, which indicates that the degree of enrichment is more significant. **B** Volcano plot of the metabolomic analysis. **C-J** The levels of main bacterial tryptophan derivatives in the gut of the control rats or MCT-induced HSOS rats, including indole-3-acetaldehyde (IAAld, **C**), indole acetic acid IAA, **D**, indole-3-propanoic acid **E**, indole-3-acetamide **F**, indole **G**, indole-3-carboxaldehyde **H**, indole-3-carboxylic acid **I** and indole-3-methyl acetate **J**. Data were shown as mean ± SEM. N = 5. *p < 0.05, **p < 0.01, ***p < 0.001. MCT, monocrotaline; HSOS, hepatic sinus obstruction syndrome

The gut microbial metabolism also changed markedly in MCT-induced HSOS rats relative to the healthy controls as the results of nontargeted metabolomics analysis. The metabolite pathway enrichment analysis demonstrated that several metabolite pathways altered significantly in HSOS rats, especially for tryptophan metabolism, then nicotinate and nicotinamide metabolism, porphyrin and chlorophyII metabolism, and tyrosine metabolism (Fig. 5A). It is well known that microbial tryptophan metabolism may produce a variety of indole derivatives, some of which have been identified as ligands for AhR [25]. In HSOS rats, levels of bacterial tryptophan derivatives in the gut were generally decreased (Fig. 5B), including the AhR ligands like indole-3-acetaldehyde (IAAld, p < 0.001, Fig. 4C) and indole acetic acid (IAA, p = 0.037, Fig. 4D). As well, indole-3-propanoic acid, indole-3-acetamide, indole, indole-3-carboxaldehyde, indole-3-carboxylic acid and indole-3-methyl acetate were also declined in the gut of HSOS rats (Fig. 4E–J), which are potential AhR ligands. It further indicated that scarcity of microbial tryptophan metabolism and ligands for AhR may play a role in the process of MCT-induced HSOS.

### Gut microbiota-derived tryptophan metabolites alleviate MCT-induced liver injury

**Fig. 6:**
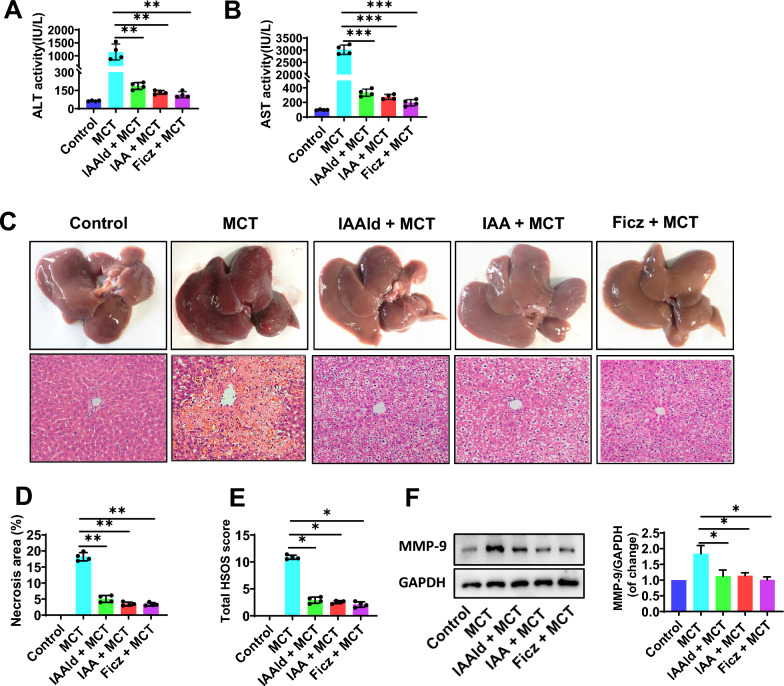
Gut microbiota-derived tryptophan metabolites alleviate MCT-induced liver injury. **A, B** Serum ALT and AST activity. **C** The typical macroscopic (upper) and histological (down) observation of liver injury. **D** The % area of necrosis quantified in a low power field (100 ×). **E** The total HSOS score. **F** The protein expression of MMP-9 in liver tissues. GAPDH was used as a loading control. Data were shown as mean ± SEM. n = 4. *p < 0.05, **p < 0.01, ***p < 0.001. MCT, monocrotaline; HSOS, hepatic sinus obstruction syndrome; IAAld, Indole-3-Acetaldehyde; IAA, Indole Acetic Acid; Ficz, 6-Formylindolo[3,2-b]carbazole; ALT, alanine aminotransferase; AST, aspartate aminotransferase; PBS, phosphate buffered saline; MMP-9, matrix metalloproteinase-9; GPADH, glyceraldehyde-3phosphate dehydrogenase

To further determine the key role of bacterial tryptophan metabolites and AhR in MCT-induced liver injury, exogenous IAAld, IAA, or Ficz (a pharmacological agonist of AhR) was administrated in MCT-treated rats. It showed that pretreatment with IAAld, IAA or Ficz alleviated the liver injury at 48 h after MCT exposure, with lower levels of serum ALT and AST (Fig. 6A, B) and lighter liver weigh and lower ratio of the liver weight and body weight (Additional file 1: Fig. S3A, B).The histological observation revealed that MCT-induced hepatic sinusoid dilatation and congestion, hepatocyte coagulation necrosis, and central lobular vein endothelial injury were significantly eliminated by IAAld, IAA or Ficz treatment (Fig. 6C), with markedly decreased % area of necrosis (P < 0.01, Fig. 6D) and total HSOS scores (P < 0.05, Fig. 6E), as well as declined expression of hepatic MMP-9 (P < 0.05, Fig. 6F).

### Gut microbial tryptophan derivatives relieve MCT-induced liver oxidative injury via the AhR/Nrf2 signaling

**Fig. 7:**
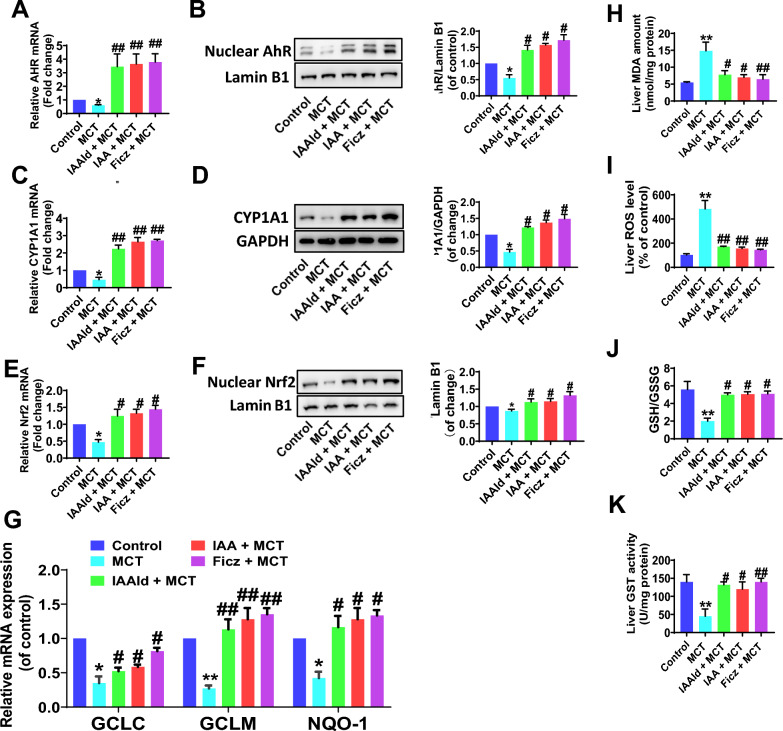
Gut microbial tryptophan derivatives relieve MCT-induced liver oxidative injury via the AhR/Nrf2 signaling. **A, B** The mRNA and protein expression of nuclear AhR in live tissues. **C-D** The CYP1A1 mRNA and protein expression of. **E–F** The mRNA and protein expression of nuclear Nrf2 in live tissues. **G** The GCLC, GCLM and NQO1 mRNA expression. **H–K** The MDA amount, ROS level, GST activity and GSH/GSSG ratio in liver tissue. Data are expressed as mean ± SEM. N = 4. *P < 0.05, **P < 0.01, vs. Control group; #P < 0.05, ##P < 0.01, vs. MCT group. MCT, monocrotaline; HSOS, hepatic sinus obstruction syndrome; IAAld, Indole-3-Acetaldehyde; IAA, Indole Acetic Acid; Ficz, 6-Formylindolo[3,2-b]carbazole; AhR, aromatic hydrocarbon receptor; CYP1A1, cytochrome P450 1A1; Nrf2, nuclear factor E2-related factor 2; GCLC, glutamate-cysteine ligase catalytic subunit; GCLM, glutamate-cysteine ligase modifier subunit; NQO-1, NAD(P)H dehydrogenase quinone 1; GPADH, glyceraldehyde-3phosphate dehydrogenase; MDA, malondialdehyde; ROS, reactive oxygen species; GSH/GSSG, glutathione/oxidized glutathione; GST, glutathione S-transferase

The expression of hepatic AhR, especially the nuclear AhR, and its target gene CYP1A1 were declined markedly in MCT-induced HSOS rats (Fig. 7A–D), which indicated the insufficient AhR activation. Supplement of tryptophan derivatives IAAld or IAA, or treated with AhR activator Ficz obviously upregulated the expression of nuclear AhR and CYP1A1 (Fig. 7A–D). Moreover, the nuclear transcription factor Nrf2 which may be directly regulated by AhR activation [9, 26] and could play a key role in the MCT-induced liver oxidative damage [27], was also raised while treating with Ficz, IAAld and IAA in MCT-induced HSOS rats (Fig. 7E, F). Furthermore, the tryptophan derivatives IAAld and IAA effectively induced activation of hepatic AhR/Nrf2 signaling, and promoted transcription of Nrf2-dependent drug-processing genes in MCT-induced HSOS rats, including glutamate-cysteine ligase catalytic subunit (GCLC), glutamate-cysteine ligase modifier subunit (GCLM) and NAD(P)H dehydrogenase quinone 1 (NQO1) (Fig. 7G). As a result, the AhR/Nrf2 activation by Ficz, IAAld and IAA further improved the MCT-induced liver oxidative damage, with the decrease of liver MDA and ROS levels (Fig. 7H–I), and the increase of the GST activity and GSH/GSSG ratio (Fig. 7J, K). All above, it indicated that gut microbial tryptophan derivatives may relieve MCT-induced liver oxidative stress injury via the AhR/Nrf2 signaling.

### Activation of AhR/Nrf2 signaling improves the LSECs damage induced by HSOS-derived fecal supernatant in vitro

**Fig. 8:**
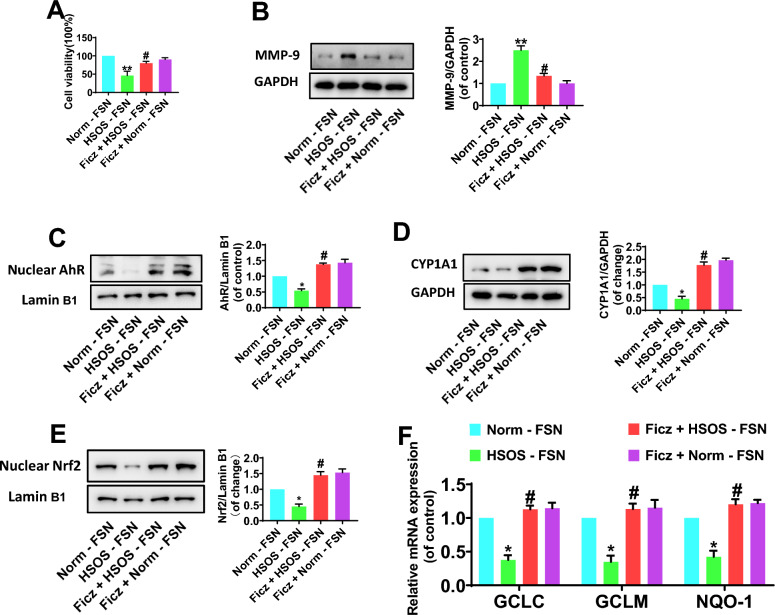
Activation of AhR/Nrf2 signaling improves the LSECs damage induced by HSOS-derived fecal supernatant in vitro. **A** Cell viability measured using Cell Counting Kit-8 assay. **B** The protein expression of MMP-9 in LSECs by Western Blot analysis. **C, D** The nuclear AhR and CYP1A1 expression in LSECs. **E** The nuclear Nrf2 expression in LSECs. **F** The mRNA expression of GCLC, GCLM and NQO1 in LSECs. Data were shown as mean ± SEM. N = 4. *P < 0.05, **P < 0.01, vs. Norm-FSN group; #P < 0.05, vs. HSOS-FSN group. HSOS, hepatic sinus obstruction syndrome; FSN, fecal supernatant; Ficz, 6-Formylindolo[3,2-b]carbazole; MMP-9, matrix metalloproteinase-9; AhR, aromatic hydrocarbon receptor; Nrf2, nuclear factor E2-related factor 2; CYP1A1, cytochrome P450 1A1; GCLC, glutamate-cysteine ligase catalytic subunit; GCLM, glutamate-cysteine ligase modifier subunit; NQO-1, NAD(P)H dehydrogenase quinone 1; GPADH, glyceraldehyde-3phosphate dehydrogenase

The HSOS-derived fecal supernatant (HSOS-FSN) induced evident endothelial injury in in vitro cultured LSECs, with decreased cell viability (Fig. 8A) and increased MMP-9 expression (Fig. 8B). The LSECs damage induced by HSOS-FSN was alleviated by activating AhR receptor with Ficz (Fig. 8A, B). Notably, HSOS-FSN led to LSECs injury which was companied with lower expression of nuclear AhR and Nrf2 (Fig. 8C, E). Apparently, Ficz promoted the nuclear AhR expression and activation in HSOS-FSN-treated LSECs, with increased CYP1A1 enrichment (Fig. 8C, D). It demonstrated that Ficz could further facilitate the nuclear translocation of Nrf2 and the expression of Nrf2 downstream genes (including GCLC, GCLM and NQO1) in HSOS-FSN-treated LSECs (Fig. 8E, F).

## Discussion

**Fig. 9:**
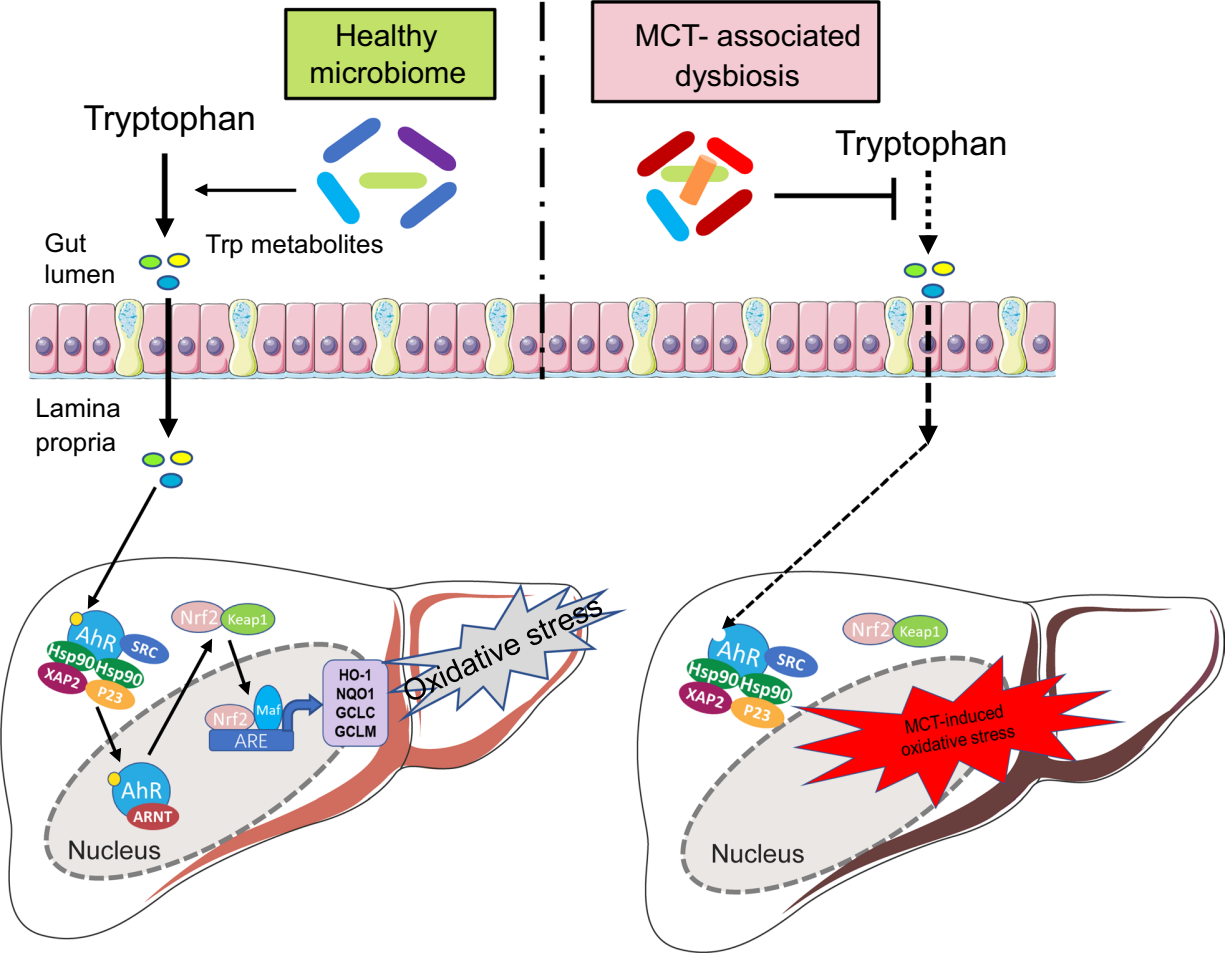
Schematic presentation of gut microbiota-derived tryptophan metabolites and AhR/Nrf2 signaling in MCT-induced liver injury. Gut microbiota-derived tryptophan metabolites, such as IAAld and IAA, could activate AhR and facilitate the nuclear translocation and activation of Nrf2 in healthy conditions, which play a role in relieving the oxidative stress injury and maintaining liver homeostasis. MCT-associated dysbiosis with decreased tryptophan-metabolizing bacteria lead to deficiency of indoles derivatives in the gut. And as a result, the AhR/Nrf2 signaling to be silenced and the liver oxidative injury to be unrestrained, thereby expedites the progress of MCT-induced HSOS. AhR, aryl-hydrocarbon receptor; Nrf2, nuclear factor E2-related factor 2; IAAld, Indole-3-Acetaldehyde; IAA, Indole Acetic Acid; GCLC, glutamate-cysteine ligase catalytic subunit; GCLM, glutamate-cysteine ligase modifier subunit; NQO-1, NAD(P)H dehydrogenase quinone 1; HSP90, heat shock protein-90; SRC, steroid receptor co-activating factor; XAP2, X-associated protein-2; ARNT, aryl hydrocarbon receptor nuclear translocator; ARE, antioxidant responsive element

In the current study, we mainly explored the critical role of gut microbiome in the pathogenesis of PAs-induced HSOS. Particularly, it demonstrated that the microbial function of tryptophan metabolism significantly declined in the gut of MCT-induced HSOS rats, with decreased abundance of some tryptophan-metabolizing bacteria, declined activity of microbial tryptophan metabolic pathway, and deficient in a series of tryptophan derivatives which are potential AhR ligands. Herein, it indicated that the inadequate microbial tryptophan metabolism in the gut and consequently a lower activity of the AhR/Nrf2 signaling in the liver may be candidate targets for the management of PAs-induced HSOS (Fig. 9)

The direct connection of the liver and the intestine through the portal vein enables the gut bacteria translocation and gut-derived products uptake to influence liver health [28]. There is accumulating evidence of decreased diversity and altered composition in gut flora in patients with various acute and chronic liver diseases [29, 30], however, the features and function of gut microbiota in HSOS-related liver injury is missing. As it shown, the fecal bacterial profiles in HSOS rats were significantly different from the healthy controls, with markedly decreased microbial diversity and altered microbial structure. Moreover, restoring the gut flora via FMT with healthy fecal microbiota could improve the MCT-induced destroy of liver sinusoidal endothelial cells and liver injury in rats. On the contrary, the rats receiving FMT with HSOS-derived fecal microbiota develop mild liver injury, and more susceptible to MCT-induced HSOS with more obvious hepatic sinusoid damage and hepatocyte necrosis. All above indicated that the gut dysbiosis may be a crucial factor affects the progression and prognosis of MCT-induced HSOS, and trying to restore the intestinal flora is necessary for the treatment of HSOS.

Here, we have also noticed that several microbiota and microbiota-derived metabolites are altered in MCT-induced HSOS rats. As it known, gut-derived products may participate in the regulation of liver homeostasis [31]. The pro-inflammatory microbial products such as lipopolysaccharide and peptidoglycan induce excessive activation of hepatic immunity and thus may aggravate the liver injury [32, 33], which may also worsen the condition of PAs-induced HSOS [12]. Instead, gut bacteria-derived metabolites such as tryptophan derivatives, bile acids, butyric acid, carotenoids and phenolic compounds may relieve the inflammatory response, oxidative stress and lipogenesis in the liver [14, 34]. The microbial metabolomics demonstrated that tryptophan metabolism is the most significantly differential metabolic pathway in HSOS rats compared with the healthy controls. Meanwhile, the tryptophan-metabolizing bacteria obviously decreased in HSOS rats, such as *Bacteroides* [35], *Bifidobacterium* [36], *Lactobacillus* [37] and *Clostridium* [38], which could generate a range of indole derivatives. It is also confirmed by the predicted bacterial functional genome based on KEGG database that the tryptophan metabolic pathway is less enriched in HSOS rats. Therefore, the declined gut microbiota-derived tryptophan metabolism may play a crucial role in the process of MCT-induced HSOS.

A series of gut microbiota-produced indole derivatives which depleted in the gut of HSOS rats were identified, such as IAAld and IAA which are ligands of AhR [25], as well as the potential AhR ligands including indole, indole-3-propanoic acid, indole-3-acetamide, indole-3-carboxaldehyde, indole-3-carboxylic acid and indole-3-methyl acetate [39]. It is reported that the *Bacteroides* and *Clostridium* can metabolize tryptophan to produce IAA [35, 38], whereas the *Lactobacilli* is able to metabolize tryptophan to produce IAAld [37], which are reduced in HSOS rats. Moreover, Supplement of tryptophan derivatives IAAld or IAA, or treated with AhR activator Ficz obviously alleviated the liver injury induced by MCT. Notably, AHR activation by microbial tryptophan metabolites were shown to improve alcohol-related liver disease [15] and non-alcoholic fatty liver disease [40]. These evidence and current results highlight the association of tryptophan metabolites and AhR with liver damage for various causes, including the MCT-induced HSOS-like liver injury.

The AhR is a ligand-inducible nuclear transcription factor that integrates dietary, microbial, metabolic, and environmental cues to maintain homeostasis [41, 42]. It is expressed in many mammalian tissues, especially in the intestine and liver [43, 44], which can be activated by a variety of small molecules, including tryptophan and indole derivatives [45]. The levels of nuclear AhR and its activated protein Cyp1A1 were markedly downregulated in MCT-induced HSOS rats, which may be associated with depleted tryptophan derivatives. Oral supplement with exogenous IAAld or IAA, or AhR activator Ficz promoted the activation of AhR and improved the liver injury in MCT-induced HSOS rats. Moreover, activating AhR by Ficz markedly alleviated the endothelial injury induced by HSOS-derived fecal supernatant in cultured LSECs, increasing the cell viability and decreasing the MMP-9 expression. It is further confirmed that to improve the deficient microbial tryptophan metabolism and to promote the activation of AhR in the liver should be an effective approach for MCT-induced HSOS.

The AhR activation may prevent MCT-induced HSOS by improving the oxidative stress liver injury. Oxidative stress is an important pathogenic event in the development of liver disease [46]. The occurrence of HSOS is related to the free radical damage to LSECs [47]. Inhibiting the hepatic oxidative stress damage could improve the prognosis of HSOS by (-)-epicatechin, chlorogenic acid, and liquiritigenin [27, 48, 49]. Herein, we also found that AhR activation by IAAld, IAA or Ficz could reduce the hepatic MDA and ROS levels, and increase the GSH/GSSG ratio and GST activity in MCT-induced HSOS, which indicated improved oxidative stress levels.

Nrf2 is the master regulator of cellular anti-oxidant responses [50], which could slow the progression of a variety of liver diseases, including viral hepatitis, liver fibrosis, drug-induced liver injury, alcoholic liver disease and non-alcoholic fatty liver disease [51, 52]. Tryptophan derivatives IAAld and IAA, also the Ficz ligand, promoted the expression and nuclear translocation of AhR, as well as the Nrf2, in the liver of HSOS rats. Meanwhile, the levels of Cyp1A1 (AhR target gene [53]), and GCLC, GCLM, NQO-1 (Nrf2 target genes [50]) were raised in IAAld and IAA treated HSOS rats, which represent the activation of phase I and phase II metabolic pathways. Notably, the transcription of Nrf2 gene can be directly regulated by AhR activation [54, 55]. AhR activation initiates the dissociation of Nrf2/Keap1 in the cytoplasm, and permits Nrf2 shuttling into the nucleus, where it binds to small Maf proteins to form a transcriptionally active complex to induce expression of anti-oxidative target genes, such as phase II-enzymes GCLC, GCLM, NQO-1^50^. Our observations thus proved the potential involvement of AhR-Nrf2 signaling in preventing liver injury induced by MCT. Gut microbiota-derived tryptophan metabolites activate the phase I (AhR-Cyp1A1) and phase II (Nrf2- antioxidative enzymes) metabolic pathways in the liver to protect the LSECs and hepatocytes from oxidative stress injury in MCT-induced HSOS.

## Conclusion

In conclusion, we showed an essential role for the gut microbiota in the context of MCT-induced HSOS, especially for the insufficient microbiome-derived tryptophan metabolism in the lumen and consequently a lower activity of the AhR/Nrf2 signaling in the liver. Accordingly, to restore or enrich the specific tryptophan-metabolizing bacteria, or to supplement with specific tryptophan metabolites (AhR ligands), or to directly administer with pharmacological AhR agonist could be potential therapeutic strategy for treating the PAs-induced HSOS.

## Supplementary Information

**Additional file 1: Figure S1.** HSOS-derived gut microbiota aggravated the liver damage induced by MCT. The relative abundance of tryptophan-metabolizing bacteria species was determined by Real-time PCR. The abundance of Bacteroides, Bifidobacterium, Lactobacillus and Clostridium was measured. Data were shown as mean ± SEM. N = 4. *p < 0.05, **p < 0.01. MCT, monocrotaline; HSOS, hepatic sinus obstruction syndrome; FMT, fecal microbiota transplantation. **Figure S2.** Fecal microbiota transplantation from normal rats restored the disordered gut microbiota in MCT rats. The relative abundance of tryptophan-metabolizing bacteria species was determined by Real-time PCR. The abundance of Bacteroides, Bifidobacterium, Lactobacillus and Clostridium was measured. Data were shown as mean ± SEM. N = 4. *p < 0.05, **p < 0.01. MCT, monocrotaline; FMT, fecal microbiota transplantation. **Figure S3.** Gut microbiota-derived tryptophan metabolites alleviate MCT-induced liver injury. **A** Liver weight; **B** the ratio of the liver weight and body weight. Data were shown as mean ± SEM. n = 4. *p < 0.05. MCT, monocrotaline; LW/BW (%), the ratio of the liver weight and body weight; IAAld, Indole-3-Acetaldehyde; IAA, Indole Acetic Acid; Ficz, 6-Formylindolo[3,2-b]carbazole.

**Additional file 2: Table S1.** List of Primers for Real-time PCR.

## Data Availability

All relevant data of this study are available from the corresponding authors upon reasonable request.
