# Supplementary figures and images for: Gut microbiota-derived tryptophan metabolites alleviate liver injury via AhR/Nrf2 activation in pyrrolizidine alkaloids-induced sinusoidal obstruction syndrome
Source: Cell Biosci. 2023 Jul 8;13:127. doi: 10.1186/s13578-023-01078-4 (PMC10329330; doi:10.1186/s13578-023-01078-4)

Supplementary Figure 1

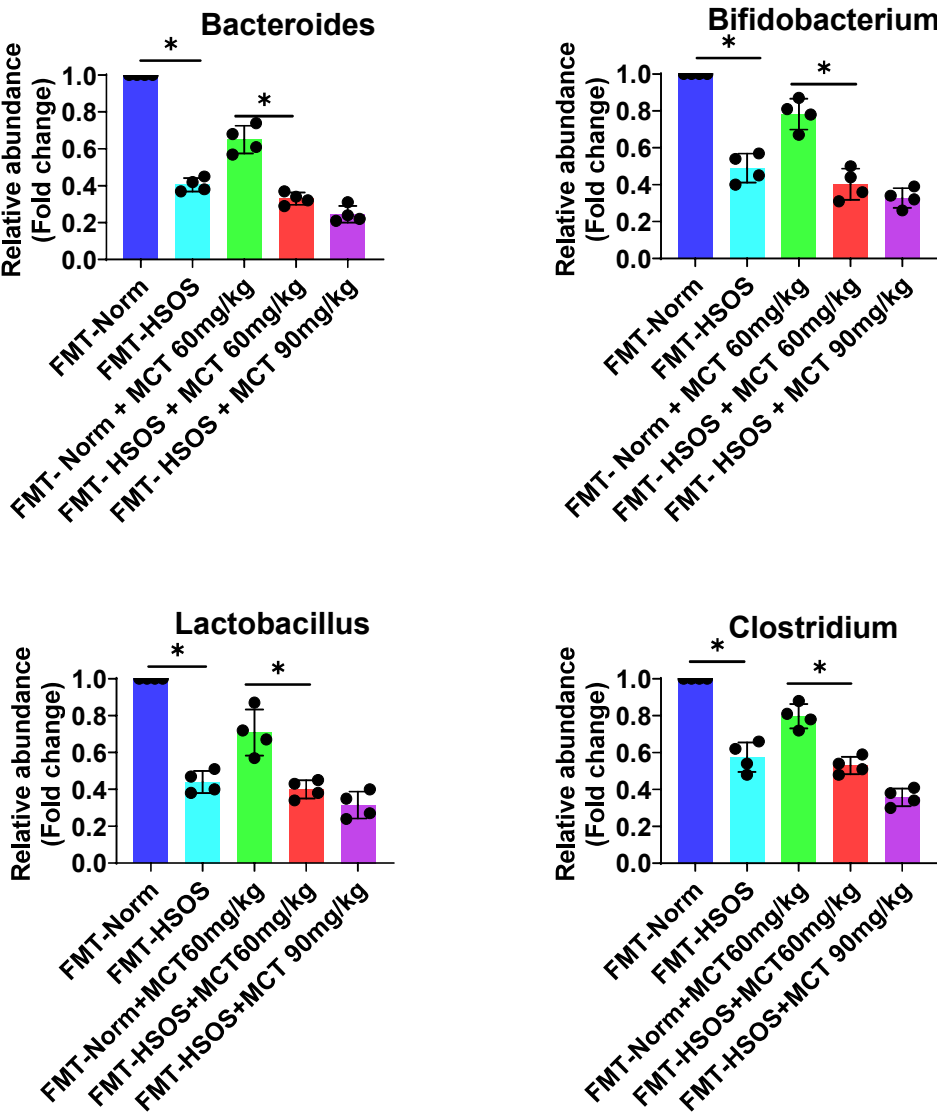

Supplementary Figure 2

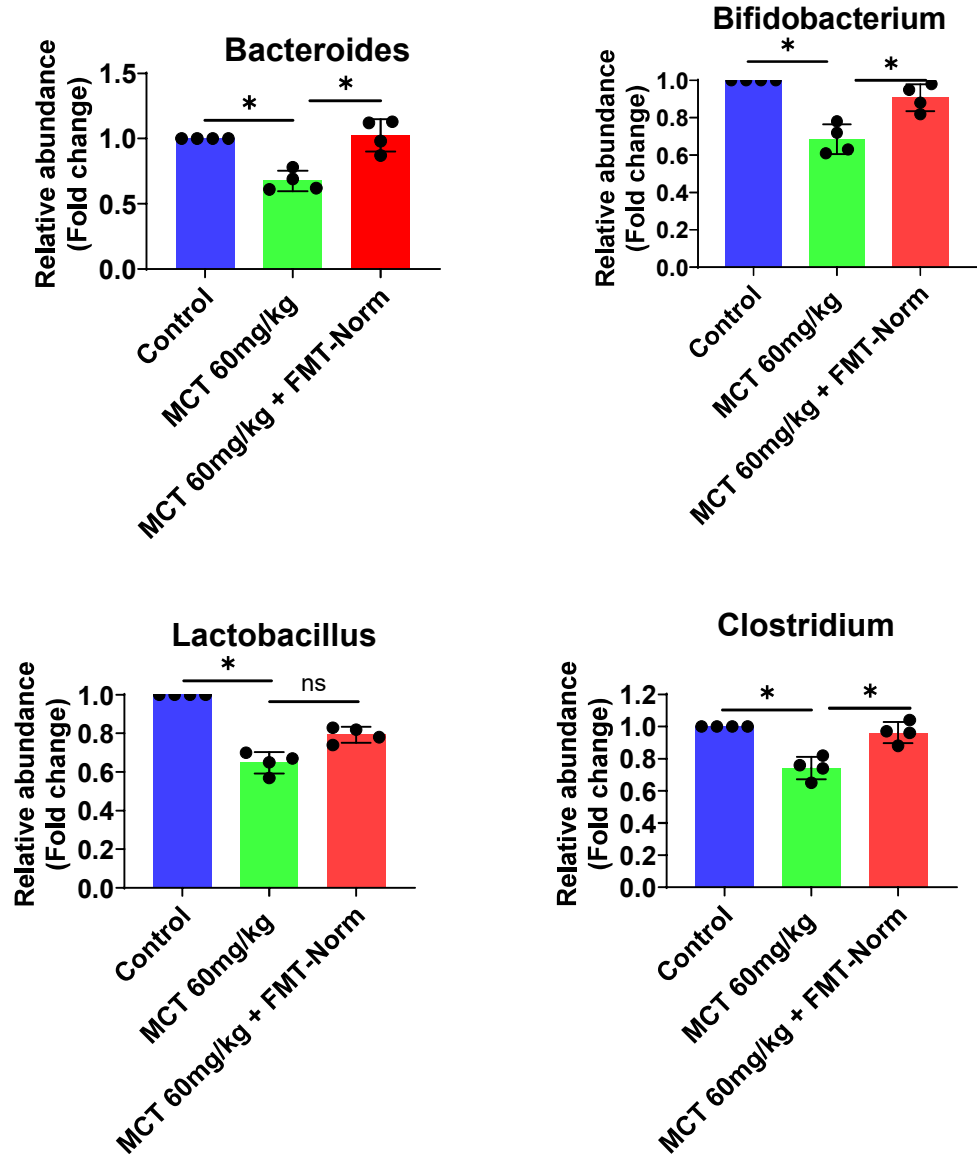

Supplementary Figure 3

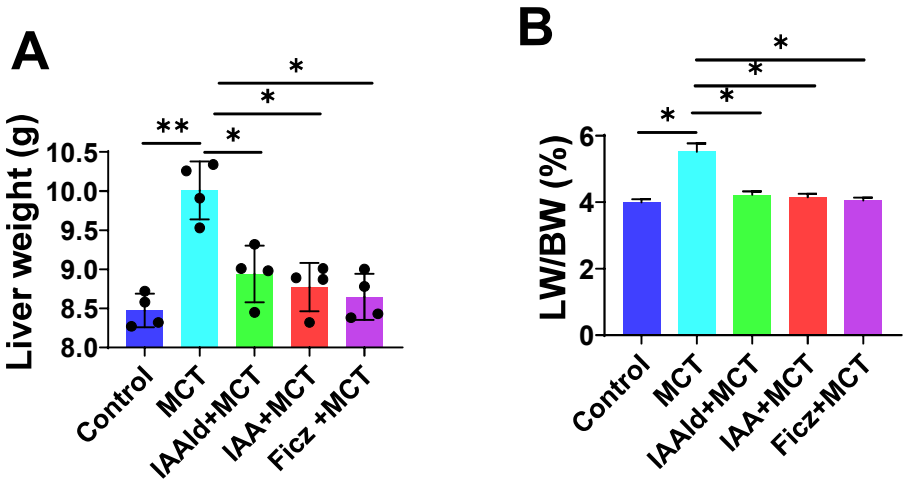

Supplement: Supplementary file 1 — Additional file 1: Figure S1. HSOS-derived gut microbiota aggravated the liver damage induced by MCT. The relative abundance of tryptophan-metabolizing bacteria species was determined by Real-time PCR. The abundance of Bacteroides, Bifidobacterium, Lactobacillus and Clostridium was measured. Data were shown as mean ± SEM. N = 4. *p < 0.05, **p < 0.01. MCT, monocrotaline; HSOS, hepatic sinus obstruction syndrome; FMT, fecal microbiota transplantation. Figure S2. Fecal microbiota transplantation from normal rats restored the disordered gut microbiota in MCT rats. The relative abundance of tryptophan-metabolizing bacteria species was determined by Real-time PCR. The abundance of Bacteroides, Bifidobacterium, Lactobacillus and Clostridium was measured. Data were shown as mean ± SEM. N = 4. *p < 0.05, **p < 0.01. MCT, monocrotaline; FMT, fecal microbiota transplantation. Figure S3. Gut microbiota-derived tryptophan metabolites alleviate MCT-induced liver injury. A Liver weight; B the ratio of the liver weight and body weight. Data were shown as mean ± SEM. n = 4. *p < 0.05. MCT, monocrotaline; LW/BW (%), the ratio of the liver weight and body weight; IAAld, Indole-3-Acetaldehyde; IAA, Indole Acetic Acid; Ficz, 6-Formylindolo[3,2-b]carbazole. [file 13578_2023_1078_MOESM1_ESM.pdf]
