# Supplementary material for: Gut microbiota-derived tryptophan metabolites alleviate liver injury via AhR/Nrf2 activation in pyrrolizidine alkaloids-induced sinusoidal obstruction syndrome
Source: Cell Biosci. 2023 Jul 8;13:127. doi: 10.1186/s13578-023-01078-4 (PMC10329330; doi:10.1186/s13578-023-01078-4)
Supplement: Supplementary file 2 — Additional file 2: Table S1. List of Primers for Real-time PCR. [file 13578_2023_1078_MOESM2_ESM.docx]

| Table S1. List of Primers for Real-time PCR. | |  |
| --- | --- | --- |
| Genes  (rat) | Forward Primer (5′- 3′) | Reverse Primer (5′- 3′) |
| AhR | CTTGTTACAGGCGCTGAATGG | CTGGAACTCAGCTCGGTCTT |
| CYP1A1 | TCTTCAGTTCAGTCCTTCCTCAC | CCACCCAGAATCCAAGGCA |
| Nrf2 | GCCCTCAGCATGATGGACTTG | TGGAGTTGCTCTTGTCTCTCC |
| GCLC | AATGGGAAGGAAGGCGTGTT | CATCCACCTGGCAACAGTCA |
| GCLM | GTTGCTATAGGCACCTCGGA | CTGGGCTTCAATGTCAGGGAT |
| NQO1 | CCGAAGCATTTCAGGGTCGT | GGGCCAATACAATCAGGGCT |
| β-Actin | AGATCAAGATCATTGCTCCTCCT | ACGCAGCTCAGTAACAGTCC |
